# Exploring understandings and approaches to decolonisation in the field of violence against women and girls: Towards conceptual clarity and actionable strategies for funding, programming and research

**DOI:** 10.1371/journal.pgph.0005664

**Published:** 2026-02-02

**Authors:** Michelle Lokot, Beatriz Kalichman, Marjorie Pichon, Ana Maria Buller, Nambusi Kyegombe

**Affiliations:** 1 Gender Violence & Health Centre, London School of Hygiene & Tropical Medicine, 15-17 Tavistock Place, London, United Kingdom; 2 Department of Preventive Medicine, Medical School, University of São Paulo, Av. Prof. Lucio Martins Rodrigues, s/n - Cidade Universitária, São Paulo, Brazil; 3 Medical Research Council/Uganda Virus Research Institute, London School of Hygiene & Tropical Medicine Uganda Research Unit, Entebbe Plot 51-59 Nakiwogo Road, Entebbe, Uganda; University of Cape Town, SOUTH AFRICA

## Abstract

Despite recognition that decolonisation is important, there remains a lack of conceptual clarity on what exactly decolonising might involve. Our qualitative study explores how colonialism and decolonisation are conceptualised within the field of violence against women and girls (VAWG), and identifies practical strategies to decolonise funding, programming and research. Our findings draw from 17 semi-structured interviews, eight focus group discussions (FGDs) and five feedback workshops (n = 83) with practitioners, researchers and donors supporting VAWG prevention and response. We identified participants through existing professional networks and used snowball sampling to recruit participants from all world regions. Interviews and FGDs were conducted in English, French, Portuguese and Spanish. We used a reflexive thematic approach for analysis. There was confusion and disagreement about what the term ‘decolonisation’ means. To address this, VAWG actors must identify the legacy and impact of colonialism for the field, and clarify the roles of Northern and Southern actors in decolonising. Our findings highlight that the area where most work is still needed is around VAWG programming, where coloniality may be embedded into programme design and implementation, requiring more concerted actions to decolonise. Funding was also identified as a key area where structural reform is needed. Research, however, was an area where there appeared to be much higher awareness and action related to decolonising, linked to momentum around dismantling research-based power hierarchies outside of the VAWG field. This research contributes to articulating the main barriers to decolonising and identifies practical actions for funding, programming and research within the VAWG field and beyond.

## 1. Introduction

In this paper we discuss the perspectives of practitioners and researchers on colonialism and decolonisation within the field of violence against women and girls (VAWG). VAWG is a global challenge, and addressing it requires multi-lateral and multi-sectoral effort, including United Nations actors, international and national NGOs, community-based organisations, service providers, researchers and grassroots actors. We seek to trace the colonial threads influencing current action to address VAWG, to explore how decolonisation is understood by actors in the field, and to identify strategies for decolonisation of the field of VAWG across funding, programming and research.

Global estimates suggest that one in three women will experience violence in their lifetime, however, between and within countries there is significant variation [1]. VAWG can take multiple forms, including domestic violence, child marriage, female genital cutting, economic abuse and coercive control. VAWG has vast consequences for individuals, relationships, communities and societies as a whole, impacting health, well-being, social relations and economic development [2,3]. The field has spent decades identifying effective prevention and responses to VAWG through research, evidence-based interventions, advocacy for legislative changes and increased funding.

In the last 5–10 years in particular, there has been increased global criticism of inequitable power hierarchies within the fields of development, humanitarianism and global health [4–7]. This includes how power hierarchies like colonialism, gender, class, race, ethnicity and education level intersect to heighten inequities [8,9]. Researchers and practitioners are increasingly reflecting on their roles in perpetuating “White Saviourism” and recognising how top-down hierarchies of engagement and intervention have been shaped by colonial ways of thinking [10–12]. This is in line with the concept of “coloniality of power” [13], which suggests colonialism is far from over, and describes how attitudes, beliefs, values and power structures that were embedded within colonialism continue to impact the world today. Coloniality is a way of thinking that perpetuates the same ideas and historical practices of colonialism, guided by the belief, whether conscious or subconscious, that Indigenous populations are “backwards and need to be modernised” based on Western standards of “acceptable” norms and behaviours [13–15]. Coloniality positions Indigenous knowledge systems as inferior [16] and is underpinned by racism [17].

Globally, there is significant recognition of how research practices are shaped by coloniality, for example, beliefs about what counts as “evidence”, what knowledge is valued, unfair authorship practices, stereotypical depictions of communities as “other” and hierarchical decision-making and funding allocation [4,12,18–24]. Within research, recognition of the impacts of coloniality has occurred across disciplines. A growing body of literature also documents practices for decolonising research, including incorporating reflexivity, rethinking what counts as knowledge and who produces knowledge, shifting decision-making to the communities to which the research pertains, using grounded methods to collect data, engaging in collaborative analysis, and ensuring fairness in who is credited for authorship [4,12,18,25–31]. Research approaches such as co-production, using equitable research principles, and feminist and decolonising methods themselves have also emerged as responses to intersecting power hierarchies [12,15,32–35].

However, programming efforts to identify and challenge coloniality have been more limited and tend to be sector-specific, with degrees of engagement varying by sector [19,36–38]. Within the field of VAWG, there is an emerging body of literature that challenges coloniality. This includes critiques of how VAWG is framed solely in terms of patriarchy, without recognition of the role of colonial powers in shaping patriarchy [12,39–42]. Scholars have urged the importance of developing strategies to tackle VAWG based on what is appropriate for the context, instead of programming responses being externally imposed [12,43–45]. They also critique stereotypical and racist depictions of VAWG that detach it from social norms and histories, urging care in how “culture” and “tradition” are described in contrast to “modernity”, and cautioning against voyeurism by those from the ‘West’ [12,43,46–51].

Within the VAWG field, while there is some recognition of how coloniality shapes research and programming, there is a lack of clarity on how decolonisation is understood, and what practical actions can be taken to address coloniality, further described in our recent scoping review of the global published and grey literature [12]. This lack of clarity is linked to the broader, global-level lack of consensus on what exactly decolonisation means in practical terms. Although decolonisation is broadly understood as describing processes of undoing/dismantling the legacies of colonialism [15,52], it is not clear what this might mean in practice. Fanon’s (1963) description of decolonisation as “unsettling” is sometimes positioned as being at odds with current approaches to decolonisation, which can come across as tokenistic or performative [53]. There is recognition that decolonisation needs to be viewed as more than a “metaphor”, involving a fundamental disruption [54]. Scholars critique the use of decolonisation as a buzzword [55] and urge researchers and practitioners to engage in more care when using this term by articulating exactly what they mean [12].

Our review identified five key strategies for decolonizing the field of VAWG: 1. Consider the context and power hierarchies within which VAWG occurs; 2. Incorporate community resources and perspectives into efforts to end VAWG; 3. Use methods and approaches to researching VAWG that centre perspectives and lived experience of communities; 4. Shift VAWG funding to local actors and ensure VAWG funding streams are more responsive to local needs and realities; and 5. Ensure local, contextually-relevant framings of feminisms inform decolonising of VAWG [12]. The current study builds on these findings, drawing on the perspectives of VAWG stakeholders to better understand how coloniality occurs within the VAWG field, how decolonisation is understood by different actors, and what additional, specific strategies may be productive for decolonising funding, programming, and research in the field of VAWG and beyond.

## 2. Methods

### 2.1. Sampling and recruitment

We identified semi-structured interview and focus group discussion (FGD) participants through our existing professional networks, the SVRI (Sexual Violence Research Initiative) Digest (a weekly email for people working on VAWG), and using snowball sampling. We used purposive sampling to maximise representation from all world regions. Eligible participants included donors, practitioners, or researchers, aged 18 years and over, working in the field of VAWG prevention or response. Interviews and FGDs were conducted in English, Spanish, Portuguese and French.

Five feedback sessions were held to discuss the initial findings from interviews and FGDs and provide further opportunities for input among participants and others working in the VAWG field. Four feedback sessions were held online with interview and FGD participants across different time zones; participants were not grouped by region. One in-person feedback session was held at the SVRI Forum in October 2024 in Cape Town, South Africa with both participants and others who expressed interest in joining the feedback sessions. Participants for the in-person session signed up using Eventbrite or were individually invited to attend, and a couple also joined on the day. As space was limited, we prioritised invitations that maximised geographic, and professional and gender representation.

### 2.2. Data collection

A total of 83 individuals participated in our study. Data collection began with semi-structured interviews in February-March 2024, that explored participant views on power hierarchies, the role of colonialism, their perceptions of decolonisation and their experiences of funding, programming, and/or research within the VAWG field. We conducted 17 interviews with 14 women and three men. Eight participants were working in institutions located in high-income countries (HIC), including Europe, Australia and the US, three were located in Asia, two in sub-Saharan Africa (SSA), two in the Middle East and North Africa (MENA), and two in Latin America and the Caribbean (LAC). A few individuals were working in regions they were not originally from.

Based on key themes identified during interviews we developed FGD topic guides, which were also semi-structured and explored the role of colonialism and decolonising the VAWG field in more depth. FGDs also explored issues related to funding that arose during interviews, and explored strategies for tackling power dynamics within research collaborations. Interviews and FGDs were conducted using Zoom and were audio recorded with participants’ informed consent. FGD participants were also invited to type in the chatbox or provide anonymous written comments using a ‘Jamboard’. We conducted eight FGDs in May-June 2024, which were mostly organised by region and area of work (Table 1). Overall, 50 women and 9 men participated; 21 were from SSA, 14 from LAC, 12 from HIC, eight from Asia and the Pacific, and four from MENA.

**Table 1:** Breakdown of focus group discussions (FGDs) and participant characteristics (n = 59).

FGD Number	Characteristics of participants	Number and gender of participants
1	Africa, programming	9; 7 women and 2 men
2	Africa, research	7; all women
3	Asia, research	6; all women
4	High-income countries, research	9; all women
5	Latin America and the Caribbean, Spanish-speaking, programming and research	9; 8 women and 1 man
6	Muiti-regional, donors	6; all women
7	Multi-regional, French-speaking, programming and research	3; 2 women and 1 man
8	Multi-regional, programming and research	9; 7 women and 2 men

We also conducted four online feedback sessions and one in-person feedback session with a total of 30 participants - seven of whom were new participants - in September-October 2024. These were not organised by region. Feedback session data were integrated into our analysis, to further nuance and deepen our analysis.

We used the Zoom automatic transcription function along with Transkriptor transcription software to create clean transcripts. Data were analysed in their original language, and quotes used in the paper were then translated into English.

### 2.3. Analysis

Our analysis was based on Braun & Clarke’s reflexive thematic analysis approach [56]. Coding was primarily inductive. We read a selection of transcripts to identify key codes and develop our coding framework. Transcripts were coded by ML, BK, and MP using Nvivo 12 software. We coded two interview transcripts together then coded the remaining individually. The same codes were used for the interviews and FGDs - except for one additional FGD code that was added to capture information on leadership of decolonisation initiatives. We made notes of key points and themes from each transcript, which were discussed during weekly meetings to enable ongoing analysis with the whole team. During writing, we continued to refine themes, and integrated key points from feedback sessions back into our analysis.

In this paper, we recognise the presence of terminology that may be “Other-ising” and reinforce assumptions about capacity and expertise. This includes “North”, “Western” and “Global South”, which, as others have argued [57], creates artificial and stereotypical divisions between actors. We recognise the problem with these binaries, but use these terms in the absence of equivalent language to signify power hierarchies, and to reflect the language used by participants. We also use the term “local” as this was used by participants, but recognise it may reinforce power hierarchies. In using this term, we do not intend to imply that “local” actors are lesser, or to diminish their roles or perspectives. We also observe the term “local” is sometimes conflated with “national” and fails to capture levels of power between actors, and recognise the importance of distinguishing between different types of actors responding in their own setting [18].

### 2.4. Ethics

This study received ethical approval from the London School of Hygiene & Tropical Medicine (LSHTM) (29 September 2022, ref: 28070). Informed written consent was obtained from all participants. Potential participants were provided with a Participant Information Sheet and Consent Form, which detailed our approach to anonymising data and managing confidentiality, to ensure they were able to make an informed decision about participation. Data was collected from 19 February 2023–24 October 2024.

### 2.5. Positionality

We sought to critically reflect on our own practice as part of conducting this study. Our team consisted of a mix of backgrounds and expertise, and we recognised the importance of placing value on different forms of knowledge throughout this project. Throughout this study we reflected together on our own experiences of VAWG research partnerships (ML, MP, BK, AMB, NK), and implementing (ML) and evaluating programmes (ML, MP, AMB, NK). We discussed how power hierarchies and privilege of different forms have shaped us individually. A few of us have direct and family experiences of colonialism (ML, NK, AMB); we have all benefited from access to higher education; we have all experienced inequities in academia related to gender and age; and multiple team members identify as being from ‘racialised minorities’ when viewed from the North (ML, NK, AMB).

We reflected on how our work experience and discipline-specific training may have predisposed us to thinking about issues in certain ways, and sought to find ways to centre and draw on Indigenous and alternative ways of knowing to counter extractive knowledge production. We met regularly to discuss our reflections on the research and especially during data collection and analysis, sought to be led by our participants’ views and experiences rather than making assumptions. During analysis and writing we sought to present participants’ views and ideas in their own words as much as possible rather than summarising or generalising. It is also important to highlight that this study was conducted at LSHTM, an institution whose power and position is linked to it being a beneficiary and contributor of British colonialism (Hirsch & Martin, 2022).

## 3. Results

This section first presents findings on how participants defined and conceptualised ‘colonialism’ and ‘decolonisation’. It then explores these terms in the context of the VAWG field, highlighting strategies for decolonisation and discussing who holds responsibility for this process.

### 3.1. Defining and conceptualising colonialism

Many different definitions of colonialism were discussed across all geographical regions. Some participants described colonialism using words like exploitation, extraction and dehumanisation. They explained that in some cases, colonialism was ongoing, for example the occupation of Palestine, extractivism in the Amazon and violence against Indigenous communities. Some also positioned colonialism as a power hierarchy that cuts across world divisions, stating: “[*C]olonialism would impact on any work that takes place across Global North and South divides*” (HIC, woman interview 11). They also reflected on how, like patriarchy, the colonial legacy, or coloniality, had been normalised”: “*it’s like, the air that we breathe*” (HIC, woman, interview 3).

The same participant went on to describe that another way colonialism was conceptualised was through the ongoing control of ideas: “*[W]e are still very much under colonial influences, in our thinking, in our dreaming, in our ideological grounding of things, in our frame of reference of the world*” (HIC, woman, interview 3). This extended to the field of VAWG, with one participant stating that colonialism led to “*assuming that people in ‘The Global South’, [and] ‘underdeveloped’ countries need to be help[ed] and do not know how to stop VAWG*” (HIC, FGD 4, Jamboard). There was also recognition that enacting colonialism wasn’t restricted to those in the Global North, and there is a need to understand country-specific inequalities and power structures that were often colonial legacies themselves: “*[W]e found that even among ourselves here in the Global South, we could also be perpetuating the same colonial approaches that we are struggling to fight*” (SSA, woman, FGD 2, P3). This lack of clarity on what colonialism is and who is perpetuating it was described by participants as adversely impacting efforts to decolonise.

### 3.2. Defining and conceptualising decolonisation

While a few participants - specifically practitioners involved in direct implementation - had never engaged with the concept of decolonisation, most participants from all regions were familiar with the term but struggled to define it. They often instead provided examples of *how* to decolonise, and emphasised the need for a “*working definition*” that would help clarify “*what it is and what it isn’t*” (Feedback session 5, in-person).

Decolonisation was sometimes defined in terms of diversity, equity, collaboration, or inclusion and was described as an overarching concept for multiple terms: “*Decolonisation is an umbrella under which we can find all these terminologies*” (SSA, FGD 1, Jamboard). However, other participants strongly emphasised that decolonisation was something different, because it seeks to identify and address colonialism and its legacy, and is linked to race:

“*[E]quity forces the power holders to think about why and how they have more privilege and power AND moreover why they don’t want to give it up. But the thing is, that could be amongst the same race or people and doesn’t address the issues of White supremacy which speaks to decolonisation.*” (Asia, woman, FGD 3, P4, chatbox)

“*Mistaking decolonisation for inclusion seems particularly like an exercise in wilful blindness, like I’m just going to pretend I don’t understand what this means…*” (Feedback session 3, online)

Among those working in the LAC region in particular, there was recognition that the decolonisation movement had originated in the Global South, while others from different regions including Asia and SSA sometimes interpreted decolonisation as something that was Western or “elite” and originating from an academic perspective (MENA, woman, interview 4).

A few participants debated whether decolonising involved structural transformation, an incremental process, or a combination of both:

*“I feel like decolonising […] you have to try to […] both dismantle or redirect or shift […] empowered institutions, and then you have to build up and strengthen institutions or systems that have traditionally been oppressed.*” (HIC, woman, interview 12)

Some participants rejected the idea of incremental change as true decolonisation, while also grappling with the practical and theoretical complexity of decolonising. This also led to some rejecting the use of the term decolonisation completely:

“*Maybe we need to think of a different word. We cannot decolonise things. It’s very difficult, [...] we have to really, you know, create a new system of how we see those things rather than bringing changes in the existing practice.*” (Asia, woman, interview 8)

Power redistribution was identified as a key component of decolonisation by many participants in the Global North and South:

“*I think that the concept of decolonising simply means taking back the power from the hands of the abuser and giving it back to the violated [...] to enable them to now have the power, the rights, the opportunities that they ordinarily were supposed to have.*” (SSA, man, FGD 1, P4)

However, participants argued that decolonisation could not fully reverse the impacts of colonialism:

“*Decolonisation is not about recovering something that has been lost; it’s not possible to recover something after so many centuries. It’s more about seeking it with the awareness that we are contaminated by colonisation—a new path based on the old, but with the understanding that we can’t return to a pure past.*” (Feedback session 1, online)

In addition to requiring structural change, decolonisation was also described by many as having a personal dimension: “*[D]ecolonisation is both a process within oneself and a relationship with oneself (...) It’s self-awareness and valuing one’s heritage*” (HIC, woman, interview 17). The idea of decolonising being both personal and political was also echoed by others: “*I guess decolonisation feels like it’s healing… And healing has to happen on so many levels of trauma*” (HIC, woman, interview 14).

### 3.3. The field of VAWG

In this section we turn to the field of VAWG, highlighting participant’s perceptions of how colonialism has impacted conceptualisations of violence, and describing two overarching decolonisation strategies. We then share specific inequalities and strategies to decolonise funding, programming, and research that arose from the data. Lastly, we share findings on perceptions of whose responsibility it is to decolonise.

#### 3.3.1. Colonial impacts on the field of VAWG.

Across all regions, many participants explained that the idea of VAWG and structures in the VAWG field are inextricably tied to a broader colonial framework, making it hard to overcome: “*the ‘nonprofit industrial complex’ is very real, and the field has been structured by capitalist and colonialist logics*” (HIC, woman, FGD 4, P5, chatbox).

They also stated that definitions and framings of VAWG were problematically “*universalised, standardised*” instead of being contextualised for specific settings (Multi-region, woman, FGD 8, P8). A health and “*disease oriented*” way of understanding VAWG was also critiqued as being narrow (Multi-region, woman, FGD 8, P8, chatbox) and playing a role in reinforcing White Saviourism: “*The language that is used […] like ‘risky behavior’ or ‘at-risk population’ that sets up a saviour dynamic*” (HIC woman, FGD 4, Jamboard).

During interviews and discussions many participants raised questions about how culture, norms and Western meanings of terms like autonomy, independence and oppression had become the standard for how VAWG interventions were framed. For example, norms programming was often primarily about “*fighting bad social norms*” with the implication that “*everything cultural is bad… every norm is negative and has to be challenged*” (SSA, woman, FGD 2, P1).

Additionally, participants across most regions discussed how notions of women’s autonomy and empowerment were sometimes positioned without the understanding of poverty as a structural force: “*[W]e’re selling them a dream and a vision, but nothing else is caught up in terms of structures and systems*” (Multi-region, woman, FGD 8, P8). The idea of women’s empowerment was mentioned by participants in LAC as “*very paternalistic*” (LAC, woman, FGD 5, P6). The Western notion of individual independence being equated to economic independence was also critiqued by a woman working in SSA as being framed as being about “*making money*,” while “*being a homemaker*” was perceived negatively despite often being important culturally (SSA, woman, interview 6).

Northern notions of well-being also reportedly shaped VAWG interventions and conceptualisations: “*[W]hat women/girls need to live well comes from the North*” (LAC, FGD 5, Jamboard), highlighting the need to base programming on conceptualisations of VAWG and needs that are articulated by those working in settings where programmes/research is occurring. For example, “Western” approaches to counselling as a response to violence was mentioned as not always suitable. In contrast, one participant suggested “local” responses to VAWG were “*presumed not to be effective*” (LAC, FGD 5, Jamboard). Some participants highlighted that the perspectives of those living in settings where programmes/research occurs were neglected, drawing attention also to how VAWG survivors’ perspectives were also not reflected in how VAWG is understood; the latter of which came out particularly from those working in low- and middle-income countries (LMICs).

#### 3.3.2. Strategies to decolonise the field of VAWG.

**3.3.2.1. Overarching strategies.** Participants across all regions consistently discussed two overarching strategies for decolonising funding, programming and research in the VAWG field. The first was carefully clarifying what decolonising might mean for the field:

“[*W]hat do we mean by decolonising? […] What are we decolonising? Is it the entire system of oppression? […] [O]r are we just trying to change [a] little bit here and there to make it look like, okay, […] we are more inclusive […] [If] we are saying that we want to take the entire White supremacy out of the entire system. Right? Is it possible?*” (Asia, woman, interview 8)

This need to clarify what is meant by decolonisation was linked to perceptions of how the term has been used as a buzzword in the sector; and a view that explaining what decolonising actually involved would ensure that it was used meaningfully.

The second strategy identified by many participants across all regions was for all actors to intentionally reflect on their own positionality, and be prepared to relinquish power and privilege. For many participants this was about questioning motives and intentions for engaging in the VAWG space: “*[A]s a privileged person, I really need to also push myself to see what I’m doing in this space. What’s my intention? What do I want?*” (Asia, woman, interview 8).

Many participants discussed how identifying power hierarchies they were positioned within was key: “*Recognising power is very important, it allows you to recognise your own privilege and be conscious of it*” (SSA, FGD 1, Jamboard). However, it was also noted by some that reflecting on privilege needed to incorporate deep, critical reflection of power going beyond a “*flippant*” acknowledgement (SSA, woman, FGD 2, P5). Beyond reflecting on power, being ready to give up power was also discussed by some across all regions:

“*How do you want to give away [that] power? […] be honest, that we are privileged […] that’s the honesty we really need from people […] who wants to support this movement.”* (Asia, woman, interview 8)

Recognition of power went beyond the individual, to also identifying ways in which the VAWG field and its institutions reproduce colonial inequalities through organisational practices in funding, programming and research.

**3.3.2.2. Strategies to decolonise funding.** Funding was the area most identified by almost all participants in FGDs and interviews as important when outlining strategies to decolonise the field. Participants critiqued how funding decisions are made from afar, resulting in agenda-setting that is often divorced from the realities of the country in question:

“*[T]he decision makers, these people are usually mostly always White men, who sit in the Washington DC offices, New York offices, you know, in the Global North, having absolutely no idea what is going on in the local context.*” (HIC, woman, interview 3)

Many participants shared different ways to decolonise funding, including through structural changes and increasing funding flexibility to make requirements less onerous. One key structural change mentioned was enabling more core funding, especially by participants working in Asia and SSA:

“*[M]any funders do not cover core costs and yet expect grantees to have strong systems and person power to ensure checks and balance. This approach makes it very difficult for grantees to implement projects comprehensively.*” (SSA, woman, FGD 1, P8, chatbox)

“*To get core funding as an organisation […] it is very, very freeing. […] It allows them to actually focus on what matters and on what they need to do [rather] than be distracted a lot by just like running after funding*”. (SSA, woman, interview 6)

The short-term nature of funding, combined with needing to follow the whims of funders, were also identified as barriers:

“*[I]nternational staff are making funding decisions and they come in cycles of four years. So you have these sort of disjointed phases of prioritisation which make it really difficult to do consistent advocacy… If a new person comes in, then suddenly it’s like, oh, actually, I want to work on disability… for example.”* (Multi-region donors, woman, FGD 6, P6)

Other structural changes recommended across multiple regions included ending international actors sub-contracting to actors working in the setting: “*[T]o deconstruct attitudes and assumptions about who can handle the money”* (SSA, woman, FGD 2, P4, chatbox). This structural shift was linked to actors implementing programmes/research in their own settings no longer being seen as solely implementing partners: “*[W]e’re not your implementing partner… You are coming alongside us to support us in this work*” (Asia, woman, FGD 3, P3). Members of an FGD conducted with researchers in Asia also discussed how “*umbrella*” funding that supported several organisations, instead of just one, could reduce “*unnecessary competitio*n” (Asia, woman, FGD 3, P6).

The need for greater flexibility within funding was also discussed as vital, spanning from applications to reporting processes. For instance, participants across all regions discussed the need to change how donors influence change agendas: “*[D]onors who come with an outcome, who come with the theory of change… With the logical framework… That’s not decolonising, that’s not giving power to…those who [are] at the grassroots*” (Asia, woman, interview 8).

Participants also advocated for allowing actors who are responding to VAWG in their own settings to write proposals in their own language. Additionally, they identified the need to reconsider proposals “*beyond the technicalities*” to ensure that it wasn’t just the “*very known*” actors who continually received funds, but also newer and smaller organisations (SSA, woman, FGD 2, P6). The need for feedback to applicants – whether successful or not – was also highlighted. Additionally, a few participants working in HIC and LAC suggested making due diligence processes easier, for example through the process of “*passporting*” (where due diligence is only done once, instead of separately for each donor) (HIC, woman, interview 12).

Improving information flows and communication between donors and recipients was also highlighted, including creating a mechanism for actors based in settings where programmes/research were occurring to provide feedback on how the partnership was working, and building platforms that gathered donors together with recipients to share feedback. This was identified as part of a shift to “*trust-based philanthropy*”, to recognise that “*no one can do this work without the other*” (SSA, woman, FGD 2, P4). Part of this trust-based approach for a few participants across multiple regions also meant improved recognition of the difficult contexts they sometimes worked within, including constrained food and job security for practitioners and researchers in countries where the VAWG programmes/research were occurring.

Lastly, the need to recognise that change takes time and success may look differently to different actors was also highlighted: “*Funders, researchers, practitioners, communities all seem to view success differently. Working towards agreeing on what we are all trying to achieve would help in deconstruction of these hierarchies*” (Africa, woman, FGD 2, P1, chatbox). One donor also suggested removing reporting requirements and instead shifting to “*requests for information*” (Multi-region, woman, FGD 6, P3). The idea was that having less onerous reporting would provide opportunities for funding to adapt dynamically to changing environments.

**3.3.2.3. Strategies to decolonise programming.** Many participants across all regions discussed the importance of shifting to programmes led by in-country practitioners to improve quality and relevance of interventions:

“*You can set up the best systems and structures and you can pour as much money as you want. But if you’re not focusing on the right things or not focusing on the way in which you can engage with people, rather than seeing it as a foreign concept or foreign agenda that’s being imposed, it won’t make a difference.”* (Multi-region, woman, FGD 8, P6)

*“[F]or local actors to be part of, from the design […] to the monitoring and evaluation processes, that’s maybe the first and the strongest way to […] have better intervention[s].”* (MENA, woman, interview 7)

Assumptions among funders and institutions from the “West” who hold funds about who has capacity (and who doesn’t) were described as influencing professional relationships and dynamics between VAWG actors; with many reflecting on how these were shaped by colonial legacies. This included the preference for hiring international staff over local staff:

*“[T]here’s nobody on this budget that is from the country that this research is happening from. So why are you trusting an American guy who has lived in [African country] for two years to be the manager for this study instead of trusting a [African country national] to do this?”* (HIC, woman, interview 12)

Differences in pay structures were also mentioned in multiple discussions, alongside the preference for international consultants: “[*T]hey pay a lot to this [international] consultant. But when you have experts in your own country, then you negotiate the payment… you don’t think that the person is enough [of an] expert*” (Asia, woman, interview 5).

Some participants working in LMICs also recommended shifting from solely assessing impact, or only focusing on numbers as part of “*positivist*” approaches (Multi-region, woman, FGD 8, P7) to also assessing programme quality: “*They just want to see that you’ve got numbers, you’ve got 300 cases coming in every month, and that’s value for money. But in terms of the holistic approach […] how is this counselling contributing to unpacking the root cause of violence?*” (Asia, woman, FGD 3, P4)

Relatedly, some also highlighted the importance of going “*beyond just addressing the outcome*” (Asia, woman, interview 8), through holistic programming that addresses interrelated, gendered structural drivers such as food insecurity and educational gaps, as well as intersectional programming that addresses, for example, violence against LGBTQI+ women or women living in humanitarian contexts.

**3.3.2.4 Strategies to decolonise research.** Many participants across regions stated that during the inception phase of research, brainstorming priorities and values to ensure shared understanding of expectations, and discussing strengths and gaps in each organisation was vital:

*“[W]hat does it mean to say you’re leading this? […] what are the kind of expectations in terms of transparency, in terms of accountability […] there’s so many small things we can do [...] If we really want to do them,”* (SSA, woman, FGD 2, P3)

Unequal power dynamics meant that real partnerships were not always possible: *“[I]t wasn’t a partnership, it was site data collectorship*” (Multi-region, woman, FGD 8, P8). To counteract this, many participants across regions suggested that frank conversations about equitable partnership should be ongoing throughout research, through regularly scheduled meetings. While these discussions could be difficult and uncomfortable, they could also be very rewarding. A few participants also stated that meeting in-person and getting to know each other informally could facilitate trust:

*“[I]t’s about creating spaces and avenues for people to know each other. You know, like be vulnerable and open to each other [… and] these uncomfortable discussions can form into very important, strong relationships that make these conversations easier to have, so that the tensions can be dealt with along the way.”* (SSA, woman, FGD 2, P3)

While most reflected on equitable partnerships between Northern and Southern organisations, one participant working in SSA also highlighted the role that larger Global South organisations could play in supporting smaller, activist-led Global South organisations through partnership and mentorship on grant applications. Along the same lines, a few participants working in HIC and SSA also emphasised considering who is best placed to conduct the research, and deferring to local, Indigenous organisations. Additionally, several participants suggested appointing Principal Investigators from the Global South, however, this also posed challenges if such countries had limited capacity:

*“[O]ne of the challenges that comes with this is […] those specific people [who have VAWG capacity] get burdened by a lot of requests and people are like, oh, you’re the Global South person.”* (HIC, woman, interview 11)

Thus, strengthening capacity was identified as an important way to prevent over-burdening experienced senior researchers. A few participants working in HIC and SSA also emphasised, however, that this should be a bi-directional exchange:

*“[W]hat this [capacity building] really means is that someone from the Global North is training or mentoring or coaching someone from the Global South. What if we approached this as a shared process? […] Because […] we have the local knowledge and the local competence […] that’s capacity.”* (SSA, woman, FGD 2, P1)

Others suggested providing more training opportunities for early career researchers in the Global South, including PhDs funded at an institution of their choice, and appropriately resourcing Global South actors, for example with high-speed internet.

Many participants across all regions stressed that during the data collection and dissemination phases, there was a need to move beyond traditional, quantitative methods, and recognise other forms of knowledge and Global South epistemologies. These included anthropological and participatory action approaches, art analysis, oral storytelling, testimonies and conversational research. Participants described how these could be combined with more commonly recognised methods, such as quantitative surveys from RCTs and qualitative research methods, to arrive at a greater understanding of VAWG, but that true decolonisation meant challenging hierarchies of knowledge:

*“[T]here’s no one form of knowledge that has greater value than another form of knowledge, but each form of knowledge has some role to play in solving this puzzle [of VAWG] at different times.”* (SSA, woman, interview 9)

Participants also suggested incorporating mixed-methods to help make more creative forms of qualitative research more ‘palatable’ to funders (HIC, woman, interview 11).

Other decolonial approaches to data collection identified by a few in HIC included ensuring participant’s autonomy in deciding whether they participate in research by explaining the research in more accessible ways. Participants stressed appropriate compensation for co-researchers beyond small “incentives” for their time (HIC, Woman, FGD 4, P5). Data co-ownership by researchers in the country was also emphasised by a participant in SSA.

Strategies to decolonise analysis and dissemination identified by many participants across all regions included investigators from the Global South being co-authors in publications, and disseminating research findings quickly to directly inform programming. A few participants described often being left out of authorship, or being relegated to a less important authorship positions:

*“There are cases where we have had to plead and beg to write papers from work we were part of…Global North collaborators don’t think of our roles beyond data collection.”* (SSA, woman, FGD 2, P1, chatbox)

Many participants suggested authorship should be clearly discussed at the beginning of projects. A couple of participants working in HICs explained that while in theory they supported co-authorship with Global South partners, in practice this could be challenging and could perpetuate tokenism: “*sometimes it does become tokenistic and kind of adding people in without them actually having been involved*” (HIC, woman interview 11).

Similarly, another participant explained that skill differences could lead to potentially unfair or performative practices:

*“[W]e’ve had early career researchers from overseas who are like they’re learning still and are supposed to lead Paper X. But then, actually, somebody from our team ends up doing like most of the work […] but then ends up being second author on it. So I feel like, that’s a situation I kind of like often run into that feels a bit unfair; like is difficult to negotiate.”* (HIC, woman, interview 2)

One solution to this proposed by several participants was that publications could be written by Global South partners in a more narrative style, to suit different formats such as book chapters or blogs. One participant stated that different dissemination formats “*helps us tell stories ethically, in such a way that you centre the voices of the people that you’re writing about*” (MENA, woman, interview 3). Others suggested academic journals could loosen their strict requirements:

*“[T]hese journals, the way you have to submit it, the way it has to be written, the specific type of referencing… it’s so discouraging […] whereas if … you’re encouraged to present other pieces… more informally written, written for lay people, or summaries or blogs… just communicating that research in different ways [is easier].”* (SSA, woman, FGD 2, P4)

Moreover, many participants stressed the importance of disseminating findings to non-academic audiences:

*“[W]e had to tell them [Global North partners], you know, the report only serves your purpose, whereas those who […] tell their stories, they actually don’t go back with anything at the end because the report is written and sits in your office. […] We have to be able to benefit from this.”* (Asia, woman, FGD 1, P3)

Sharing findings in languages accessible to research participants and to broader audiences was also consistently discussed in FGDs and interviews. One participant working in a HIC suggested accessible dissemination could also be promoted through conferences in the Global South, and co-organised by Indigenous organisations, highlighting challenges regarding conferences:

*“[If] people cannot travel where they need to travel, you can’t even begin to share knowledge that is diverse.”* (HIC, woman, interview 17)

#### 3.3.3. Who should lead efforts to decolonise.

When asked about who should lead decolonisation efforts, responses were mixed, and were not split along geographic lines. Some participants said those who experienced colonisation would be better positioned to formulate appropriate responses:

*“[T]hose who are colonised should actually be the ones taking lead. And I tend to think that they should be able to have discussions and reflections on what was there before the colonial process and see those positive things which were washed away during the colonial process and see how to bring them back. In that way, we shall be actually making use of the Indigenous knowledge to reduce violence against women and violence against girls.”* (SSA, woman, FGD 2, P2)

*“You came, you colonised me, and now you’re talking about decolonising… You’re taking everything away from me and you’re still coming to say you want to take, you want to correct.”* (SSA, woman, FGD 1, P5)

Others said that those based in the Global North should be responsible for decolonising since they exercised power over others. However, most participants saw decolonisation as a collective responsibility:

*“It is everyone’s responsibility, you can’t just say it’s the people who are oppressed who have to do the work.”* (Feedback session 3, online)

*“Anyone that has a little bit of power should do something.”* (Multi-region, woman, FGD 6, P3)

*“We need to put our efforts together, everybody has to help. All of us can contribute with something different, all levels. If we take someone out, we might miss something.”* (Feedback session 2, online)

Participants across all regions and roles also made a distinction between taking a leadership role in decolonisation, versus being accountable, responsible or supporting decolonisation:

*“It’s like this whole question of, do we work with men and boys to end violence? […] [T]hey are the beneficiaries from patriarchy. […] Do we work with them to […] blindly, […] take over the agenda… Or do we work with them with accountability? And what does accountability look like?… That’s exactly the same for me with the question on decolonisation.”* (Multi-region, woman, FGD 6, P1)

*“I like the distinction between leadership and who needs to be responsible or perhaps accountable [...] I think those parties, including countries, institutions, whatever, those probably should be the ones at least financially sponsoring the decolonisation part on violence against women and girls.”* (HIC, woman, FGD 4, P4)

However, some participants questioned if accountability was possible, asking, “*How do you hold someone accountable?*”, and arguing this is difficult in practice and unclear how it could be enforced (Feedback session 5, in-person).

## 4. Discussion

To our knowledge, this is the first study that explores how the concepts of colonialism and decolonisation are framed and operationalised within the VAWG field, and what practical strategies could contribute to decolonisation. We contribute important insights into how practitioners and researchers grapple with decolonisation, including strategies that might help the VAWG field better consider how to decolonise funding, programming and research. Moreover, some of our findings are not only relevant to the VAWG field, but have broader applicability.

Although much of the debate among participants focused on how decolonisation is framed and applied, we also found that colonialism as a concept may be defined differently by different actors, and that identifying the role and impact of colonialism for VAWG is critical to how decolonisation is framed [38]. Our study reveals blurred lines between who is deemed the coloniser and who is the colonised, suggesting the North-South binary is less helpful [57] because of what Quijano [13] terms the “coloniality of power” - the mindsets, values, beliefs, attitudes, and economic and political structures of colonialism that remain a legacy in the world today. Coloniality continues to shape the interactions and intersecting power hierarchies structuring society, which participants revealed has profound impacts on multiple aspects of efforts to prevent and respond to VAWG: how funding and agenda-setting occur, the assumptions made about who has expertise and who doesn’t, the gender norms that shape society, relationships between actors, how VAWG is defined and measured, how interventions from the North are implemented wholesale, extractive and positivist data collection, unfair authorship practices and problems in how the lives of those who have experienced violence are represented by those outside the context.

While the findings about colonialism’s ongoing impacts on research practices are not new [4,19–22,24,58], our study draws attention to how coloniality impacts VAWG programming. There is some literature on how coloniality shapes programme design (e.g., [41]), however, our findings highlight systematic patterns of coloniality infusing other aspects of programming beyond design alone. This includes how VAWG is defined, how concepts like empowerment and culture are positioned, and how paternalistic assumptions about those who are “Other” [10,49] shape how interventions are designed and implemented. We suggest much needs to change within the VAWG field to correct these unequal power hierarchies that are embedded within VAWG programmes. Feedback from practitioners in our study suggests there is appetite to do programming differently and we argue this is an area where the VAWG field should focus its efforts, given that shifts to research practices are in many cases already underway and have the benefit of drawing on lessons from other sectors and disciplines. VAWG programming remains a unique space where more work might be needed for practitioners to identify and call out coloniality, and where most change is needed. We also suggest VAWG funding structures and processes require significant reform as they are the area where the majority of participants across all regions felt coloniality continues. Funding structures and processes are not unique to the VAWG field, so reforms in this area would have wide-reaching impacts.

Similar to our review findings, we found mixed understandings of what decolonisation involves, and concerns about the concept becoming a buzzword [12,55], which have broader relevance beyond the field of VAWG. While a few participants felt decolonisation is akin to other concepts like equity and inclusion, many others stressed that what was distinct about decolonisation is the central focus on colonialism as a power structure, and that this core meaning was vital to retain. We found that some perceived colonialism as an academic or elite concept, while others stressed how the decolonial project was grounded in activism and Indigenous scholarship, suggesting more work is needed to define and ground this concept within the decolonising movement. This may require more reflection on the most appropriate terminology for communicating the intention of decolonisation, including translating terms into local languages. Within these reflections about terminology, it is also crucial to maintain a focus on action, rather than defining concepts alone. Participants had mixed opinions about who should lead decolonisation efforts, suggesting the need to clarify not only what decolonising involves, but also who is responsible for which aspects of decolonising. While there is value in seeing decolonising as a collective task, it may still be helpful to differentiate how various actors can contribute to this overarching task, while also holding that the North-South binary is not always helpful in articulating who holds power and who needs to relinquish it.

We found that although participants were able to articulate practical decolonising strategies - particularly in the case of funding structures - they also caveated some of their suggestions by observing tensions that need to be considered before these strategies are implemented (summarised in Table 2), many of which have relevance beyond the field of VAWG. For example, reflecting on positionality and power was often discussed, but participants also flagged how positionality can be used simplistically to cover over complex dynamics. Others have also found positionality statements may be used to centre whiteness or emphasise expertise [38]. Shifting power and roles such as authorship to local/national actors is also not straight-forward, as it may reinforce tokenistic or performative inclusion, and unless capacity-strengthening is done intentionally from the outset, can increase burdens on actors implementing VAWG programmes or research in their own settings. As others have also reflected, these practitioners may not wish to lead authorship or may not have the time for this, and it cannot be assumed that authorship is something that is always desired [12,59]. As we found in our study, challenging hierarchies of evidence might mean producing less academic outputs, which requires fundamental shifts in how different forms of knowledge are valued. Further, capacity-strengthening efforts may also be constrained by real, institutional and structural barriers that limit the ability of actors working within the country in question to meaningfully participate in leading research or programming processes, such as working in conflict settings or having limited access to electricity and internet [31,60]. The recommendations for practical strategies and tensions associated with these strategies are discussed below in Table 2.

**Table 2:** Summary of participant’s strategies, tensions and considerations for decolonising the Violence Against Women and Girls (VAWG) Field.

OVERARCHING/CROSS-CUTTING STRATEGIES	
**Strategy**	**Tensions and considerations**
1. Clarify what decolonising actually means (sections: 3.2; 3.3.2.1)	Name colonialism, racism, White Supremacy and other systems to ensure clarity about what decolonising might mean. Recognise the opportunities and limitations in seeing decolonising as fundamentally unsettling versus something that is addressed incrementally - or a combination of both.
2. Reflect on positionality and relinquish power (section 3.3.2.1)	Critically assess and discuss motivations, power hierarchies, and privilege influencing involvement in funding, programming and research. Move beyond acknowledging privilege to identifying its impacts and taking meaningful action to reduce the harms of power hierarchies. Consider professional facilitation for navigating discussions about power within the team.
**FUNDING STRATEGIES**	
**Strategy**	**Tensions and considerations**
1. Change funding structures (section 3.3.2.2)	Shift funding terms to be more flexible and partnership-driven. Cover core costs of actors working within the country. Simplify due diligence processes (e.g., ‘passporting’). Ensure funding is flexible, accounts for complexity and builds in time for stakeholders to invest in building relationships. End subcontracting by international actors by enabling direct funding to local organisations. Introduce ‘umbrella funding’ models covering multiple organisations. Consider trust-based philanthropy approaches to address power hierarchies between funders and recipients.
2. Fund holistically (section 3.3.2.3)	Avoid setting rigid funding agendas; allow stakeholders to define priorities. Consider structural VAWG drivers (e.g., economic conditions, infrastructure) rather than narrowly framed solutions. Support programmes and research based on Indigenous knowledge systems rather than only evidence-based or positivist models. Recognise that change takes time and that efforts to shift VAWG programming may not always result in immediate quantifiable changes.
3. Reconsider funding criteria and application processes (section 3.3.2.2)	Change how calls for proposals are done, allowing longer timeframes and for submissions to be provided in multiple languages. Seek not just ‘technical’ information in proposals, but expand ways of conceptualising knowledge and capacity to valuing non-tangible aspects, such as in-country networks. Move beyond funding the typical, known actors but be willing to fund newer, smaller organisations. Provide detailed feedback to all applicants, so that they know how to improve in the future.
4. Reduce reporting burdens on grantees (section 3.3.2.2)	Shift from requesting overly-onerous submission of reports, to using simpler reporting processes.
5. Invest in improving relationships with grantees (section 3.3.2.2)	Create ongoing mechanisms to receive feedback from grantees, including from those involved in sub-contracts. Create platforms and opportunities to bring all grantees together for experience-sharing and providing feedback to donors.
**PROGRAMMING STRATEGIES**	
**Strategy**	**Tensions and considerations**
1. Change how implementing partners are viewed in the partnership (section 3.3.2.3)	Recognising that implementing partners are equal partners is vital to decolonising.
2. Ensure actors living within the countries/communities of interest lead programme design (section 3.3.2.3)	Avoid situations where external actors impose ideas of what works or define what interventions look like. Create space for actors living and working within countries/communities to lead on programme design based on their expertise and knowledge of the context.
3. Shift from focusing on numbers when defining impacts (section 3.3.2.3)	Recognise that quality of programmes is also important and seek to track this instead of solely quantitative impacts.
4. Ensure holistic programming (section 3.3.2.3)	Ensure interventions consider how to address structural drivers, including livelihoods, social safety nets, food insecurity and educational gaps.
**RESEARCH STRATEGIES**	
**Strategy**	**Tensions and considerations**
1. Ensure team expectations and roles are clearly defined from the outset (section 3.3.2.4)	The inception meeting of the research project is a critical opportunity to identify expectations and clarify roles.
2. Create opportunities to invest in relationships across the partnership (section 3.3.2.4)	Informal, social activities can be an important way to bridge gaps and invest in relationships.
3. Support smaller activist organisations through partnership and mentorship (section 3.3.2.4)	Larger organisations can play an important role in supporting smaller, grassroots actors.
4. Consider who is best placed to conduct research (section 3.3.2.4)	Reflecting on whether research is needed and who should conduct/lead this work is a vital part of decolonising. At times, taking a step back to defer to Indigenous actors or those living/working within the country to lead research may be what is required.
5. Ensure Principal Investigators are located in the Global South, when possible (section 3.3.2.4)	Considering who in the Global South organisation can lead the research and act as Principal Investigator can help with decolonising traditional research partnerships. It may be challenging to identify Principal Investigators with VAWG capacity, requiring research teams to go beyond the researchers/organisations who they typically collaborate with, or at times to revisit if the research should occur at all without local leadership. Discussions need to happen about whether Global South actors want to lead or have the time and capacity for taking on leadership.
6. Discuss publications and authorship early (section 3.3.2.4)	Ensure conversations about authorship are upfront and include Global South partners from the outset. Ensure all authors are clear on what is required from them. Don’t impose authorship but rather, recognise not everyone values being a named author. Avoid tokenistic authorship practices, such as adding people as authors without them being involved in writing - which can be performative. Authorship practices should involve ongoing honest conversations so they more fairly reflect who does the work. Discuss what types of outputs and publications are relevant to the different people involved in the partnership and consider non-academic outputs
7. Capacity-strengthening as a bi-directional process should be prioritised (section 3.3.2.4)	Approaching capacity-strengthening as a mutual, bi-directional process can help tackle assumptions about who has capacity and who doesn’t. Effort needs to be made to avoid over-burdening Global South actors by requiring them to develop and deliver training. Training early career researchers in the Global South can help set up future research partnerships. Part of capacity-strengthening might mean acknowledging the resource barriers faced by researchers in the Global South, such as limited availability of high-speed internet.
8. Draw on other ways of knowing (section 3.3.2.4)	Methods should move beyond traditional approaches to considering how to use Indigenous, anthropological and participatory action, story-telling, testimonial and conversational research approaches. Effort needs to be made to challenge hierarchies of knowledge and the emphasis on positivist approaches, especially the positioning of quantitative data as more valuable or stronger evidence than qualitative data. Consider how not just quantitative methods, but qualitative and mixed methods research can deepen the level of analysis on VAWG.
9. Improve informed consent processes and value participants’ time (section 3.3.2.4)	Ensure consent processes are not overly onerous and appropriate for participants who are less comfortable with reading and writing. Provide incentives and benefits to thank participants for participating in research, recognising the time burden this places on them.
10. Ensure data and findings are accessible (section 3.3.2.4)	Enable joint ownership of data between research partners Publish findings in languages that are accessible to participants and through a range of methods (e.g., blogs, podcasts, webinars, etc.) beyond traditional academic papers or reports and in alternative styles (e.g., narrative styles). Consider conferences that are more accessible and less costly.

Our study had a few limitations. Ensuring participation from a wide range of actors proved challenging. Although we used a multi-level sampling strategy and aimed for geographical diversity and a balance of practitioners, researchers and donors, geographical location alone does not guarantee diverse perspectives, as many participants work in settings different from those they are originally from. While we conducted the data collection in multiple languages (English, Spanish, Portuguese and French), our ability to ensure participants could contribute in languages comfortable to them was constrained by the language capacities of the research team. Reaching practitioners actively engaged in service provision was also difficult due their limited availability. This was a particular issue for participants working in the MENA region, as data collection coincided with the escalation of conflict in Gaza, further constraining their ability to take part.

## 5. Conclusion

This study contributes important insights about how colonialism and decolonisation are framed and operationalised within VAWG funding, programming and research, with broader implications for the field of VAWG and beyond. Our study identifies the need to clearly articulate what constitutes coloniality within the field of VAWG, and also to clarify what decolonising means in practice. The inconsistent understanding of these terms has led to sometimes contradictory perspectives on who should be responsible for decolonising, suggesting that work needs to be done to position decolonisation as a collective task rather than one assigned to Global “North” or “South” actors - not least because these binaries can be reductive and lack operational value. We found that while recognition of how coloniality shapes research and funding is well-understood by VAWG actors and reflects broader conversations within other fields like public health, an important gap for the VAWG field is further clarifying how coloniality shapes VAWG programming.

In outlining key strategies that might be helpful in approaching decolonisation of the VAWG field (see Table 2), we found changes to funding to be a key structural reform which is pivotal in shaping power hierarchies. Our findings highlight that decolonising is by no means straight-forward, as other context-specific, capacity-related and practical considerations also impact efforts to transform funding, programming and research. Moreover, decolonisation is not something that can be achieved and assumed to be complete, rather it is an ongoing process for all actors. We hope that the strategies emerging from this work contribute a helpful next step in the process to decolonise the fields of VAWG, public health and beyond.

## Supporting information

S1 Checklist Inclusivity in global research. (DOCX)
